# HIV-related travel restrictions: trends and country characteristics

**DOI:** 10.3402/gha.v6i0.20472

**Published:** 2013-06-03

**Authors:** Felicia Chang, Helen Prytherch, Robin C. Nesbitt, Annelies Wilder-Smith

**Affiliations:** 1 Institute of Public Health, University of Heidelberg, Heidelberg, Germany; 2 Program of Emerging Infectious Diseases, Duke-NUS Graduate Medical School, Singapore; 3 Lee Kong Chian School of Medicine, Nanyang Technological University, Singapore

**Keywords:** HIV/AIDS, travel restrictions, restrictions on entry, stay and residence, migrants, mobility

## Abstract

**Introduction:**

Increasingly, HIV-seropositive individuals cross international borders. HIV-related restrictions on entry, stay, and residence imposed by countries have important consequences for this mobile population. Our aim was to describe the geographical distribution of countries with travel restrictions and to examine the trends and characteristics of countries with such restrictions.

**Methods:**

In 2011, data presented to UNAIDS were used to establish a list of countries with and without HIV restrictions on entry, stay, and residence and to describe their geographical distribution. The following indicators were investigated to describe the country characteristics: population at mid-year, international migrants as a percentage of the population, Human Development Index, estimated HIV prevalence (age: 15–49), presence of a policy prohibiting HIV screening for general employment purposes, government and civil society responses to having non-discrimination laws/regulations which specify migrants/mobile populations, government and civil society responses to having laws/regulations/policies that present obstacles to effective HIV prevention, treatment, care, and support for migrants/mobile populations, Corruption Perception Index, and gross national income per capita.

**Results:**

HIV-related restrictions exist in 45 out of 193 WHO countries (23%) in all regions of the world. We found that the Eastern Mediterranean and Western Pacific Regions have the highest proportions of countries with these restrictions. Our analyses showed that countries that have opted for restrictions have the following characteristics: smaller populations, higher proportions of migrants in the population, lower HIV prevalence rates, and lack of legislation protecting people living with HIV from screening for employment purposes, compared with countries without restrictions.

**Conclusion:**

Countries with a high proportion of international migrants tend to have travel restrictions – a finding that is relevant to migrant populations and travel medicine providers alike. Despite international pressure to remove travel restrictions, many countries continue to implement these restrictions for HIV-positive individuals on entry and stay. Since 2010, the United States and China have engaged in high profile removals. This may be indicative of an increasing trend, facilitated by various factors, including international advocacy and the setting of a UNAIDS goal to halve the number of countries with restrictions by 2015.

According to the UNAIDS report of 2012, globally 34 million (31.4–35.9 million) people were living with HIV at the end of 2011 (1). Worldwide, an estimated 0.8% of adults aged 15–49 years are living with HIV, although the burden of the epidemic continues to vary considerably between countries and regions (1). Improved global access to effective antiretroviral therapy (ART) is enabling more persons living with HIV (PLWHIV) to live longer and have more productive lives (1). Increasingly, PLWHIV engage in international travel. Information on the exact numbers, demographics, and motivations of PLWHIV who cross international borders is limited, but it suggests that mobility is common and is of increased importance (2–4). At the same time, the complexity of mobile population dynamics between countries and regions is also increasing. These dynamics may also have an impact on the epidemiology of HIV (2, 4, 5).

Travel restrictions for HIV-positive individuals were generally adopted by governments in the early years of the epidemic when little was known about the disease and when there was great fear regarding its spread (6–8). Governments believed that controlling national borders could protect the health of their citizens by preventing the spread of the virus into the country and/or could moderate financial and systematic costs for treatment, care, and support of HIV-positive foreigners (6–10). Previously, the restrictions were termed ‘HIV travel restrictions’; however, due to the growing awareness that their scope reaches far beyond travel, they are now commonly referred to as ‘HIV-related restrictions on entry, stay, and residence’ (7). Hereafter, they will be referred to as ‘HIV-related restrictions’ or simply ‘restrictions’. The focus of this study is on restrictions that regulate entry, stay, or residence in a country solely on the basis of HIV status. These are typically manifested as laws or administrative instructions that require people to indicate their HIV-free status before entering or to remain in a country (10, 11). Table 1 summarizes the general procedures and potential outcomes of the restrictions.

**Table 1:** Summary of the features of HIV-related restrictions at different stages of the travel or immigration process and the outcome of testing positive for the HIV virus

Phase and location	Requirement	Outcome if the person tests positive for HIV
Pre-departure in country of origin	The visa applicant must submit to an HIV test before or at entry.	The applicant is excluded from entry. *and/or* The person's positive status is recorded on the passport and/or on some other immigration document, form, or record (removable or permanent depending on the country).
On arrival at entry point to destination country	The visa applicant must declare his/her HIV status upon entry or show negative results of an HIV test.	The applicant is excluded from entry. *and/or* The person's positive status is recorded on the passport and/or on some other immigration document, form, or record (removable or permanent depending on the country).
In the destination country	Person must be tested for HIV in order to renew the visa or permit (frequency depends on the country).	The person is put into immigration detention pending deportation. *and/or* The person is deported. *and/or* The person's positive status is recorded on the passport and/or on some other immigration document, form, or record (removable or permanent depending on the country).

Adapted from the UNAIDS Report of the International Task Team on HIV-related Travel Restrictions. Geneva 2008 (10). These outcomes can be applied individually or in combination.

Testing for HIV status under these circumstances differs greatly from testing or screening for health promotion and disease prevention purposes that are intended to identify and benefit persons who test positive (12, 13). Testing associated with HIV restrictions has been compared to mandatory testing, compulsory or conditional screening, and prohibitive pre-employment testing (10–12, 14–16). International organizations and public health professionals have historically opposed these restrictions, stating that they are impractical (6, 13) and ineffective (6), they promote stigma and discrimination (7), and violate human rights (7, 12).

HIV restrictions are a multidisciplinary and transnational issue (17, 18). The lack of easily available information on such restrictions has reduced the likelihood of HIV-positive populations being forewarned about them (2, 10). Travel medicine practitioners often lack information to provide up-to-date advice to travelers. Migrant workers may face denial of entry.

Our aim was to describe the situation of such restrictions as of 2011 in a comprehensive overview for travel medicine practitioners and public health professionals who work with mobile populations. In addition, we sought to understand and describe the geographical distribution of countries with restrictions, to identify and examine the trends of such restrictions over time, and to investigate the characteristics of the countries concerned.

## Methods

We analyzed 193 World Health Organization (WHO) member states as defined in June 2011 (19). Countries were grouped according to the WHO regional categories (20). Reports published by the Joint United Nations Programme on HIV/AIDS (UNAIDS), International Task Team on HIV-related Travel Restrictions, and Human Rights and Law Teams were also used to identify countries with restrictions and the nature of the restrictions (21–23). The countries identified in these documents were based on information from the Global Database on HIV-Specific Travel & Residence Restrictions (24) that governments were given the opportunity to verify (23). A world map template was modified to display the countries with restrictions.

We selected nine indicators based on their considered relevance to the topic of HIV restrictions. All data were extracted from already standardized data sets from reputable organizations: Joint United Nations Programme on HIV/AIDS (UNAIDS), United Nations Development Programme (UNDP), United Nations Department of Economic and Social Affairs (UNDESA), World Health Organization (WHO), World Bank, International Labour Organization (ILO), Transparency International and Ministries of health (25–31). Data from more recent years (2009–2010) were taken in preference to data from earlier years. Where pre-dated data were used, a note was made in the dataset.

The following indicators were investigated to describe the country characteristics: population at mid-year (in thousands), international migrants as a percentage of the population, Human Development Index (HDI), estimated HIV prevalence (age: 15–49), presence of a policy prohibiting HIV screening for general employment purposes (Yes/No), government and civil society responses to having non-discrimination laws/regulations which specify migrants/mobile populations (Yes/No), government and civil society responses to having laws/regulations/policies that present obstacles to effective HIV prevention, treatment, care, and support for migrants/mobile populations (Yes/No), Corruption Perception Index (CPI), gross national income per capita, and the Atlas method (current US$).

All available data were imported from Microsoft Excel to STATA statistical software (Version 12.0; Stata Corporation, College Station, Texas, USA) for analysis between groups of countries ‘with restrictions’ and ‘without restrictions’. Data for GNI per capita and HDI were further classified according to World Bank and UNDP categories (32, 33).

We compared countries with HIV restrictions to countries without restrictions using χ^2^ tests for categorical variables, and Mann–Whitney U tests for continuous variables.

## Results

**Fig. 1:**
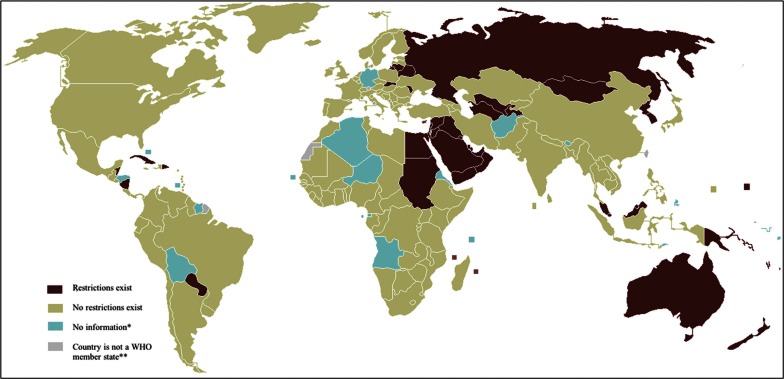
Status of HIV-related restrictions on entry, stay, and residence in 193 member states of the World Health Organization in June 2011. *Countries with no information were not reported in the UNAIDS survey (Algeria, Angola, Cape Verde, Equatorial Guinea, Eritrea, Niger, Sao Tome and Principe, Seychelles, Bahamas, Bolivia, Honduras, St. Vincent and the Grenadines, Suriname, Afghanistan, Germany, Bhutan, Timor-Leste, Cook Islands, Kiribati, Nauru, Niue, Palau, Tuvalu). It is uncertain whether these member states were checked for restrictions and evidence was inconclusive, or whether they were not checked for restrictions during the survey. **This includes associate members; states and member states with observer status and diplomatic recognition.

**Fig. 2:**
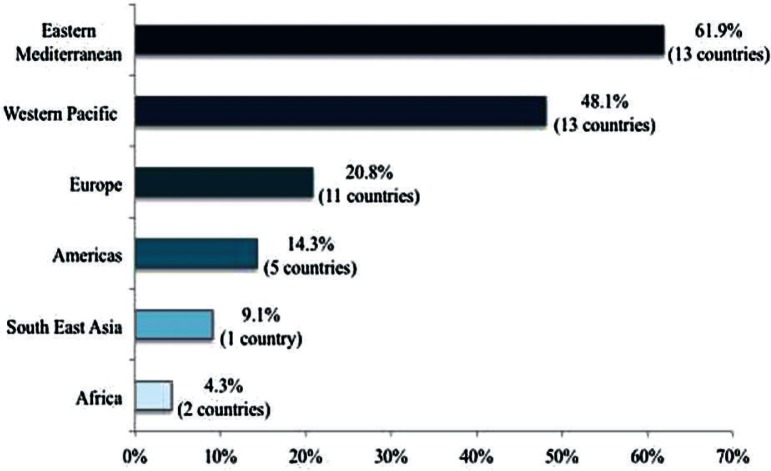
Intraregional percentages for the distribution of the 43 countries with restrictions. Percentage of countries within each region with restrictions, denominator is the total number of countries in each region.

Restrictions were present in 45 of 193 WHO countries (23%; Fig. 1). The majority of the countries (37 countries, 82%) with restrictions were found in the Eastern Mediterranean, Western Pacific, and European Regions. The Eastern Mediterranean has the highest percentage of countries with restrictions (62%) when taking into consideration the total number of WHO countries in the region (Fig. 2). Fifty-eight percent of the countries with restrictions were in the high or very high category on the Human Development Index, 32% were in the medium category, and 11% were in the low category. Thirty-three percent of the countries with restrictions were in the high category by income, 20% were in the upper middle category, 40% were in the lower middle category, and 7% were in the low category.

Of the countries with restrictions, it was further noted that only 21 countries provide clear information regarding the stage of the travel or migratory process at which the verification of status is needed. Five countries (1.1% of all WHO countries with restrictions) require declaration of HIV status or discretionary approval (including waivers) for entry/any length of stay, four countries (0.09% of all WHO countries with restrictions) deny applications for entry by HIV-positive people for stays as short as 10 days up to 90 days, and 21 countries (47% of all WHO countries with restrictions) deport foreigners once HIV-positive status is known.

Table 2 shows countries that have made changes to restrictions between 2008 and 2011. Of the countries that removed restrictions, four were from Europe (Armenia, Georgia, Poland, and Ukraine), three were from the Americas (United States of America, Ecuador, and Panama), two each were from the Western Pacific (China and Micronesia) and Southeast Asia (Bangladesh and India), and one each from Africa (Namibia) and the Eastern Mediterranean (Tunisia).

**Table 2:** Countries that were removed or added to UNAIDS listings between 2008 and 2011

Status*	Countries	Region
Eliminated (confirmed)	Armenia	Europe
	China	Western Pacific
	Namibia	Africa
	United States of America	Americas
Removed (elimination unconfirmed)	Bangladesh	Southeast Asia
	Ecuador	Americas
	Georgia	Europe
	Micronesia	Western Pacific
	Panama	Americas
	Poland	Europe
	Tunisia	Eastern Mediterranean
	Ukraine	Europe
	India	Southeast Asia
Added (unconfirmed)	Mauritius	Africa

* Confirmation criteria: an official government, UNAIDS or Global Database statement was issued. A country that is ‘Removed (elimination unconfirmed)’ is one that has been taken off the UNAIDS list but confirmation was not found.

Tables 3 and 4 summarize the characteristics of countries with HIV-related restrictions. Countries with restrictions had a lower median mid-year population, lower estimated median HIV prevalence (age: 15–49), and a higher median percentage of the population as international migrants than countries without HIV-related restrictions. A higher proportion of countries without restrictions also had policies prohibiting HIV screening for general employment purposes than countries with restrictions.

**Table 3:** Quantitative indicators in countries with and without HIV-related travel restrictions

	With restrictions		Without restrictions		
			
Indicator	*n*	Median (IQR)	*n*	Median (IQR)	*p*†
Population (1000), 2010	45	5177 (1297–21512)	123	10277 (3169–33797)	0.029
International migrants as percent of population (%), 2010	45	4.2 (2.0–22.4)	123	2.9 (0.2–10.0)	0.018
Human Development Index, 2010	43	0.70 (0.64–0.83)	122	0.70 (0.48–0.79)	0.069
HIV prevalence in adults 15–49 (%), 2009*	31	0.1 (0.1–0.5)	109	0.5 (0.1–1.8)	0.002
GNI per capita, Atlas method, 2010 (US$)	43	5030 (2340–18730)	123	4509 (1050–12660)	0.290
Corruption Perception Index, 2010	40	3.4 (2.4–5.6)	118	3.3 (2.4–4.6)	0.992

† *p*-value from Mann–Whitney U-test comparing medians;

* countries with HIV prevalence as <0.1 were included with a HIV prevalence as 0.01.

**Table 4:** Qualitative indicators in countries with and without HIV-related restrictions

Indicator		With restrictions, *n* (%)	Without restrictions, *n* (%)	Overall	*p*†
Policy prohibiting HIV screening for general employment purposes	Yes	15 (17.4)	71 (82.6)	86	0.031
	No	20 (32.8)	41 (67.2)	61	
Government response: non-discrimination laws or regulations	Yes	11 (16.2)	57 (83.8)	68	0.039
	No	24 (30.8)	54 (69.2)	78	
Civil society response: non-discrimination laws or regulations	Yes	15 (21.1)	56 (78.9)	71	0.312
	No	21 (28.4)	53 (71.6)	74	
Government response: laws, regulations or policies that present obstacles	Yes	9 (31.0)	20 (69.0)	29	0.430
	No	27 (23.9)	86 (76.1)	113	
Civil society response: laws, regulations or policies that present obstacles	Yes	12 (24.0)	38 (76.0)	50	0.925
	No	22 (24.7)	67 (75.3)	89	

† Chi-square *p*-value.

## Discussion

HIV restrictions are present in 23% of WHO countries. Overall, there appears to have been little change in the total number of countries with restrictions in the past 20 years. Previous reports estimate 50–60 countries with restrictions in 1989 and 1991 (6, 34), and 17 countries in Europe in 2010 (35), which is comparable to our findings. Given this apparently small overall variation in the past 20 years, the removal of 13 countries between 2008 and 2011 is a notable change. However, it is difficult to make concrete statements regarding trends in the number of countries with restrictions, due to the ambiguity of definitions (6, 23, 24), and lack of data (21–23). Despite this, some observations can be made that may provide insights into the developments related to the removal of these restrictions. The first is that two of the countries that removed the restrictions are highly influential at the international level: the United States and China. The second is that the elimination of the recent restrictions has been publicly praised by key actors, such as the UN Secretary General, and is seen as an example for other countries to follow (36). The third is that the removal of the restrictions has increased in importance on the international HIV and AIDS agenda. This is illustrated by the UNAIDS strategic goal for 2015 that calls for: ‘HIV-related restrictions on entry, stay and residence eliminated in half of the countries that have such restrictions’ (17). The fourth is that countries in every region removed restrictions in the past 2 years, with the only new restriction found in the African Region.

The removal of restrictions by the United States stemmed from the country's position as a leader in the global response to HIV and AIDS, and also intense domestic advocacy (http://www.aber.ac.uk/en/media/Rushton). The Chinese government first stated its intention to remove its ban at the 2008 International AIDS Conference, although this was not confirmed until April 2010, shortly after the change in the stand of the United States had been enacted, and in advance of the 2010 World Expo held in Shanghaiz.

The analysis also indicated substantial variation in regional distribution of the countries with restrictions. In absolute numbers, the Eastern Mediterranean, Western Pacific, and Europe had the most countries with restrictions. In the Eastern Mediterranean and Western Pacific regions, the proportions of countries with restrictions (62 and 48%, respectively) were greater than half of those in other regions. This may be indicative of similarities in regional contexts that may influence the persistence of such restrictions.

Although the categorization by specific types of restrictions varied considerably, the majority of countries had deportation restrictions rather than restrictions for entry or short-term stays. This finding supports previous reports (3, 9, 10) that the populations most affected are non-nationals who wish to relocate permanently, reside or stay in destination countries for longer periods of time such as labor migrants and expatriates. However, deportation may be more highly documented for several reasons: Testing is often part of the procedure for the re-issuing of residency, employment, or study permits (9–11, 37) and routine and consistent implementation may facilitate identification or make such policies more difficult to conceal. There is also potential for more severe outcomes when it comes to deportation – such as death during confinement (11, 38) prior to repatriation.

We found that 89% of the WHO countries with the highest proportions of international migrants (as a percentage of the population) have travel restrictions. This is important information for public health authorities dealing with migrant populations. The relationship between HIV restrictions and higher migrant populations may plausibly be attributed to several factors: When migrants constitute a large population, states may be faced with greater public opinions on issues such as xenophobia, stigma, and discrimination in decision making (39). Countries with higher percentages of international migrants may face conflicts due to dependency on migrant labor or tourism (14). Of the 10 countries with the highest proportions of international migrants, six are within the Eastern Mediterranean region (Qatar, United Arab Emirates, Kuwait, Jordan, Israel, Oman, and Saudi Arabia), one in the Western Pacific (Singapore) (40). In terms of absolute numbers, two of the top 10 countries, Russian Federation and Saudi Arabia, have restrictions (40). Asia and the Pacific have the second highest number of tourists and migrants after Europe (41). Governments of such countries may be pressured into adopting more stringent laws/restrictions to exclude foreign workers (13, 42) to address national concerns on foreign influences and appease voters (14). Rigorous policies may also be due to the fear of changing cultures, particularly in regions such as the Eastern Mediterranean and Western Pacific that have remained fairly isolated in terms of global cultural exchange (7, 42).

An analysis of the legal environment indicated that the absence of protective policies, laws, or regulations was more often found in countries with restrictions. The figures showing the presence of legislation aimed at protecting the human rights of PLWHIV reveal that this remains a low priority for most countries, despite the known importance for responding to the HIV/AIDS epidemic (43). According to UNAIDS, HIV-related restrictions on entry, stay, or residence are often a proxy indicator for high levels of discrimination against PLWHIV (1). HIV-related travel restrictions undermine the progress toward reducing stigma and discrimination experienced by HIV-seropositive individuals and violate international agreements such as the *Declaration of Commitment on HIV/AIDS*, *Political Declaration on HIV/AIDS*, and the *Siracusa Principles* (10). Arguments have been made that HIV restrictions force PLWHIV to find ways to bypass formal systems (7, 9), may be counterproductive (44), discourage people from being tested (45), or delay infected individuals from seeking treatment (44). These impacts can be long-term as illustrated in Sweden where the fear of deportation was one of the main factors associated with late testing among migrants (45).

The lack of association with GNI per capita suggests that the economic capacity of a country does not appear to influence the decision of governments to impose HIV restrictions. Our findings showed that very few countries with restrictions had a low GNI per capita, and the median GNI between countries with and without restrictions were not significantly different. This would appear to refute the economic arguments made by proponents for the restrictions.

Our findings also showed that countries with lower HIV prevalence tend to opt for HIV travel restrictions, possibly because they perceive themselves to be more vulnerable to the introduction of HIV. Furthermore, more countries with smaller populations have HIV restrictions than countries with larger populations. Perhaps HIV-related restrictions are perceived to be more important in countries with small populations because a small absolute increase in the number of PLWHIV will be proportionately larger than for countries with larger populations. The impact may be larger in the context of limited economic and health services capacity (8, 46, 47) to absorb HIV-positive non-nationals into the health system and perceptions of high-risk behaviors by non-nationals and PLWHIV (48). This underlies the importance of continued advocacy by health professionals on the limitations of such restrictions with a view to restrictions being reversed.

## Conclusion

Our study showed that there are still a substantial number of countries with HIV restrictions for entry, stay, and residence, despite recent removal of such restrictions from key countries. Health practitioners working with mobile populations are well placed to advise and educate individuals who may be affected by these restrictions. Impacts on individual health include but are not limited to: increased risk of interrupted adherence to ARV medication (2, 3, 5), increased risk of deportation and detainment that has implications with regard to reduced access to treatment (38), and risk of psychological stress in travel/immigration process (45). HIV restrictions not only have direct implications on individual health but also influence the structural factors affecting HIV and AIDS for mobile populations. These restrictions may contribute to stigma and discrimination (7), increase the lost opportunities for treatment and prevention when persons at risk of infection or already infected with the virus avoid formal systems (15, 45) and deter progressive developments such as the design of culturally and linguistically appropriate clinical and public health services (49). As the elimination of these restrictions becomes of increasing importance on the global HIV and AIDS agenda, health practitioners, particularly those dealing with travel and migrant issues, should be involved in the discussions and the complex contexts surrounding the restrictions.
